# Seasonal Changes in Sleep Duration in African American and African College Students Living In Washington, D.C.

**DOI:** 10.1100/tsw.2007.128

**Published:** 2007-06-12

**Authors:** Janna Volkov, Kelly J. Rohan, Samina M. Yousufi, Minh-Chau Nguyen, Michael A. Jackson, Courtney M. Thrower, John W. Stiller, Teodor T. Postolache

**Affiliations:** ^1^ District of Columbia Department of Mental Health and St. Elizabeths Hospital, Residency Training Program, 2700 Martin Luther King Jr. Avenue, S.E., Washington, D.C. 20032, USA; ^2^ Psychology Department, University of Vermont, John Dewey Hall, 2 Colchester Avenue, Burlington, VT 05405, USA; ^3^ Mood and Anxiety Program, Department of Psychiatry, University of Maryland School of Medicine, 685 West Baltimore Street, MSTF Building, Room 502, Baltimore, MD 21201, USA

**Keywords:** seasonality, ethnicity, seasonal affective disorder, sleep duration, college students, biological night

## Abstract

Duration of nocturnal melatonin secretion, a marker of “biological night” that relates to sleep duration, is longer in winter than in summer in patients with seasonal affective disorder (SAD), but not in healthy controls. In this study of African and African American college students, we hypothesized that students who met criteria for winter SAD or subsyndromal SAD (S-SAD) would report sleeping longer in winter than in summer. In addition, based on our previous observation that Africans report more “problems” with change in seasons than African Americans, we expected that the seasonal changes in sleep duration would be greater in African students than in African American students. Based on Seasonal Pattern Assessment Questionnaire (SPAQ) responses, African American and African college students in Washington, D.C. (N = 575) were grouped into a winter SAD/S-SAD group or a no winter diagnosis group, and winter and summer sleep length were determined. We conducted a 2 (season) × 2 (sex) × 2 (ethnicity) × 2 (winter diagnosis group) ANCOVA on reported sleep duration, controlling for age. Contrary to our hypothesis, we found that African and African American students with winter SAD/S-SAD report sleeping longer in the summer than in the winter. No differences in seasonality of sleep were found between African and African American students. Students with winter SAD or S-SAD may need to sacrifice sleep duration in the winter, when their academic functioning/efficiency may be impaired by syndromal or subsyndromal depression, in order to meet seasonally increased academic demands.

## INTRODUCTION

From a biological standpoint, sleep duration is determined by an interaction between the circadian pacemaker and the sleep homeostat[1]. Seasonal changes in sleep have been described in both clinical and normal populations[2,3,4,5]. Seasonal changes in sleep duration are most pronounced among individuals with sleep and mood disorders[6,7,8]. Numerous studies have found a higher incidence of depression with hypersomnia during the winter months than in other seasons[4,5,9]. A pattern of recurrent winter hypersomnia has also been described[10]. Individuals with winter-type seasonal affective disorder (SAD)(depressive episodes during the fall and winter months that remit in the spring) tend to sleep longer when depressed than those with summer-type SAD (i.e., depressive episodes during spring and summer months)[4,7,9].

The seasonality of sleep observed in mood- and sleep-disordered populations is also present in the general population, albeit to a lesser degree. In an epidemiological survey of Maryland residents, 92% of contacted individuals reported seasonal changes in sleep duration and 27% considered it a noticeable problem[2]. The average reported sleep duration during winter was 7.41 h compared to 7.05 h during summer, and those who endorsed a winter mood pattern (i.e., reported “feeling worst” in winter) reported even longer sleep duration in winter (7.61 h)[2]. Although most studies indicate more extreme seasonal sleep changes in SAD patients than in the general population[2,3,4], one study found a similar increase in sleep duration during the fall and winter in both groups[8].

Sleep duration is related to the duration of the “biological night” as programmed by the circadian pacemaker and defined by neuroendocrine markers, such as the duration of nocturnal melatonin secretion[11]. In a recent study, the duration of biological night was determined to be greater in long (≥ 9 h/night) than in short (≤ 6 h/night) sleepers[11]. In another study, melatonin secretion (a cardinal marker of biological night) was prolonged in winter compared to summer in SAD patients, but not in healthy controls[12].

Another factor associated with the degree of seasonal changes in sleep duration is geographic latitude. Results from a population study at four U.S. latitudes found that Florida residents reported less seasonality of sleep than Maryland, New York, or New Hampshire residents, and that Maryland residents reported less than New Hampshire residents[3]. Residents of more northern regions, including Norway and countries north of the polar circle, experience more depression with hypersomnia than depression with insomnia during the winter[13]. A population survey in Japan suggested greater winter-related changes in sleep duration in more northern cities compared to more southern cities[14]. Conversely, in African countries where seasonal variation in photoperiod is limited due to proximity to the equator, studies of college students suggest no seasonality in sleep patterns[15,16]. In addition, Caucasians who relocate to Africa demonstrate a nonseasonal sleep pattern similar to the native African population[17].

College students, as in the population at large, may exhibit seasonality in their sleep duration. For example, college students in Maine demonstrated a general tendency to sleep longer during the winter months[18]. Sleep duration in college students is particularly important because of academic pressures. As adolescents and young adults around the world get insufficient amounts of sleep during the week, they report feeling sleepy during the day and try to compensate by sleeping late on weekends[19,20], which further results in circadian phase delay and a “jet-lag-like” state during the first days of the week.

Sleep is problematic in college students. Of particular importance, college students often engage in poor sleep hygiene behaviors and demonstrate marked variability in their sleep schedules[21]. Hectic academic and social schedules, along with other common student practices (e.g., excessive caffeine intake and alcohol/substance abuse), contribute to significant sleep disturbances, a problem that may be escalating. Over the past 30 years, the average daily sleep duration has decreased by more than 1 h in the student population[22]. Academic demands have a marked seasonal variation. Academic performance, study habits, and test-taking abilities may all be affected by seasonal changes in sleep. Students who have relocated to more northern latitudes to attend school may be particularly vulnerable to seasonal sleep changes. A better understanding of seasonality of sleep in college students may lead to better interventions that can ultimately result in improved academic performance.

The purpose of the present study was to compare self-reported sleep duration during winter vs. summer in a large sample of African and African American college students in the Washington, D.C. metropolitan area. Based on the finding that biological night, which is related to sleep duration, is longer during winter than summer in patients with SAD, but not in healthy controls[11], we hypothesized that students who met survey criteria for winter SAD or S-SAD would report sleeping longer in winter than in summer, and that this difference would be significantly greater than in the group with no winter diagnosis. Although there are no studies comparing African American and African students on seasonal variation in sleep duration, we hypothesized, based on our previous observation, that Africans would report more "problems" with change in seasons than African Americans[23], and that Africans would experience more pronounced seasonality of sleep than African Americans.

## METHODS

## Participants

Participants were African and African American undergraduate and graduate students at four academic institutions in the Washington, D.C. metropolitan area, including three colleges/universities and one nursing school. The protocol was approved by the Institutional Review Board of the D.C. Department of Mental Health. Students signed an informed consent after a full description of the study. Inclusion criteria were (1) identifying oneself as African or African American and (2) a minimum of 2 years residency in the greater Washington, D.C. area.

## Measures

Sleep duration was reported on the Seasonal Pattern Assessment Questionnaire (SPAQ)[24], administered once either in spring or fall. The SPAQ is a widely used, self-report instrument for assessing seasonality; the sleep duration–related question is formulated as "Approximately how many hours of each 24-h day do you sleep during each season (including naps)?" For this study, our dependent measures included winter sleep length and summer sleep length.

In prior studies, the SPAQ has been used to estimate SAD and S-SAD prevalence and computes a global seasonality score (GSS)[2]. SAD requires the following: (1) GSS $\geq 11$, (2) a positive response to the question "If you experience changes with the seasons, do you feel that these are a problem for you?", and (3) rating the severity of the problem as "moderate", "marked", "severe", or "disabling" in degree. According to Rosenthal et al.[24], the S-SAD category is ascribed when (1) GSS = 8 or 9 or (2) GSS $\geq 11$ and the respondent considers seasonal changes to be nonproblematic or only a mild problem. In order to qualify for winter-type SAD or S-SAD, the respondent must endorse feeling worst in December, January, and/or February in response to the question "At what time of the year do you feel worst?" and meet the above criteria. Although the SPAQ does not discriminate well between SAD and S-SAD, the SPAQ has demonstrated good sensitivity, specificity, and positive predictive value when the categories of SAD and S-SAD are combined into a "winter problem group"[25]. The SPAQ has demonstrated high test-retest reliability in college students[18].

### *Demographic Information*

A brief questionnaire was used to assess demographic and other background information (age, gender, place of birth, length of stay in U.S., level of education) in a classroom setting at their respective institutions. African or African American ethnicity was defined as identifying oneself as an African or as an African American.

### Statistical Analyses

Using a mixed 4-factor within-subjects design where season is the within-subjects factor and sex, ethnicity, and seasonality are between-subjects factors, we conducted a 2 (season: winter, summer) × 2 (sex: male, female) × 2 (ethnicity: African, African American) × 2 (winter seasonal pattern: winter SAD/S-SAD diagnosis, no winter diagnosis) ANCOVA on reported sleep duration, controlling for age.

## RESULTS

### Demographic Characteristics

Of the original sample of 820 students recruited for the study, 216 were excluded due to missing data on one or more of the variables in the analysis (i.e., winter sleep length, summer sleep length, ethnicity, sex, or age). An additional 29 participants were excluded because of inaccurate reporting of sleep duration (26 reported sleep duration ≤ 3 h, three reported sleep duration ≥ 24 h per day). The final sample of participants used in these analyses included 575 students (437 African Americans and 138 Africans). The sample was predominantly female (62.8%), had a mean age of 29.52 years (SD = 9.03; range = 17–67), had spent an average of 19.81 years (SD = 12.41) in the U.S., and obtained a mean GSS on the SPAQ of 8.60 (SD = 5.28). Regarding winter diagnostic groups, 13.5% of students met SPAQ criteria for winter SAD or S-SAD. Prevalence estimates for SAD and S-SAD in this sample have been reported elsewhere[23,26].

### Winter and Summer Sleep Length

Age-adjusted means for winter sleep duration and summer sleep duration are presented in Table 1 by sex, ethnicity (i.e., African American or African), and winter diagnosis group (i.e., winter SAD/S-SAD or no winter diagnosis). The repeated measures ANCOVA on sleep duration revealed no significant three- or four-way interactions. The ANCOVA, however, did yield a significant season × winter diagnosis group interaction, Wilks' lambda $F$ (1, 566) = 7.73, $p = 0.006$, and a season × age interaction, Wilks' lambda $F$ (1, 566) = 18.84, $p < 0.001$. Specifically, after adjusting for age, the winter SAD/S-SAD group reported a significantly shorter sleep duration (by 21.0 min) in the winter than in summer, $t(77) = –2.15$, $p = 0.035$, and the no winter diagnosis group reported sleeping 24.6 min longer in the winter than in the summer, $t(496) = 6.57$, $p < 0.001$.

## DISCUSSION

To our knowledge, this is the first study of seasonal changes in sleep in a non-Caucasian sample in the U.S. (i.e., African American and African students) and the largest study to focus on seasonality in sleep duration in university and college students. Our results in students who did not meet criteria for SAD is consistent with prior studies in this geographic region that found a longer sleep duration in the winter than in the summer among both the general population and individuals with winter SAD[2,3,4]. However, surprisingly, in students who met criteria for SAD or S-SAD, sleep duration was shorter in winter as compared to summer.

Our study is consistent with recent efforts focused on sleep and wakefulness in adolescents and young adults[27,28,29,30,31,32,33]. For instance, the largest difference between preferred sleep and actual sleep duration have been reported in adolescents[32]. In a recent study, all adolescents reported having at least one symptom of sleep disturbance (mostly having nonrestorative sleep), while 25% of all adolescents of different ethnic groups (European Americans, African Americans, and Mexican Americans) reported

**TABLE 1**
**Age-Adjusted Means for Winter Sleep Duration and Summer Sleep Duration, by Sex, Ethnicity, and Winter Diagnosis Group**

| | Winter Sleep | Summer Sleep |
|---|---|---|
| Winter SAD/S-SAD | | |
| African Americans | | |
| Females | 7.642 (0.312) | 8.029 (0.249) |
| Males | 8.070 (0.473) | 8.245 (0.377) |
| Africans | | |
| Females | 7.220 (0.652) | 8.059 (0.519) |
| Males | 6.720 (0.922) | 6.259 (0.734) |
| No Winter Diagnosis | | |
| African Americans | | |
| Females | 7.878 (0.134) | 7.460 (0.106) |
| Males | 7.708 (0.178) | 7.353 (0.142) |
| Africans | | |
| Females | 7.760 (0.252) | 7.290 (0.201) |
| Males | 7.616 (0.276) | 7.173 (0.220) |

*Note:* All values are displayed as mean hours (SEM).

symptoms of insomnia on a daily basis[27]. No significant ethnic differences have been reported in symptoms of insomnia between European, African, and Mexican Americans in this study[27]. Similarly, ethnicity had also not been a factor in morning and evening preferences of the studied population in New Zealand in adults between 30 and 49 years old[29].

However, other studies suggest an effect of ethnicity on sleep and sleep disturbances. For instance, African American young and middle age adults have been reported to have increased light sleep, decreased deep sleep, increased sleep latency, and a higher rate of respiratory problems[30]. Adolescents living in the U.S. originating from Europe have been reported to have a higher rate of insomnia as compared to adolescents originating in China and Central America. Adolescents from Africa, Central America, and Mexico reported having increased rate of hypersomnia[28].

In young adults, sleeping fewer hours is associated with poorer health[33]. As reduced sleep duration had been previously associated with weight gain, probably via reduced leptin and elevated ghrelin[34], it may be of relevance that a reduced duration of sleep in the winter coincides with seasonal weight gain[35]. Our results add to the increasing knowledge on seasonality in children and young adults who are predominantly students[23,26,35,36]. One important limitation of our study was not collecting information about socioeconomic status, as effects of ethnicity and socioeconomic status are compounded and difficult to tease apart. For instance, multiple awakenings, difficulties falling asleep, and chronic sleep problems are more frequent in the unemployed[31], while the degree of the contributing factors of depression and stress remain unknown.

Certainly, national origin rather than ethnicity may have an important contribution on seasonality. For instance, in a study that evaluated the relationship between seasonality and “eveningness-morningness”, students of Italian origin, but not those of Spanish origin, had higher incidences of S-SAD in evening types compared to morning types[37].

. Considering the well-established seasonality of indoor and outdoor aeroallergens, it is important to note that it has been shown that sleep, daytime functioning, and mood changes are affected by inflammatory mediators and congestion connected to allergic rhinitis[38,39]. In fact, mood sensitivity to high pollen counts has been associated with a self-reported greater seasonality of mood[40]. The differences in sensitivity and exposure to indoor and outdoor allergens in African and African American students have not been systematically studied, and may result in a greater seasonality of mood, sleep duration, and sleep quality. Treatment with corticosteroids that improves nasal congestion provides significant relief of breathing difficulties, improving quality of sleep in patients with allergic rhinitis[39].

Finding that the students who met SAD criteria sleep shorter in winter as compared to summer was unexpected, as SAD patients, but not normal controls, have been reported to have prolonged biological nights (i.e., melatonin secretion) in winter compared to summer[12]. There are several plausible explanations for this surprising finding. Previous research suggests that an individual's habitual sleep duration is related to the ability to tolerate the homeostatic pressure to sleep[41]. It is possible that some individuals, under certain circumstances, are able to tolerate the pressure to sleep in order to accomplish important goals. Students with winter SAD or S-SAD may experience a decrement in their ability to perform academically in the winter when symptoms are manifested (e.g., concentration difficulties, low energy, low motivation) and, therefore, require more time to accomplish their schoolwork successfully (i.e., increased time spent studying for exams resulting in less time for sleep). In order to compensate for this effect, the recourse of students is usually an increased intake of alerting pharmacological agents, most commonly caffeinated beverages.

Another possibility for the finding of shorter sleep duration in students with winter SAD/S-SAD is the ethnic composition of the sample. Our sample was comprised of African American and African students of both sexes, and our winter diagnosis group included both SAD and S-SAD. In contrast, in the study in which patients had a shorter duration of melatonin secretion than controls in summer compared to winter, the participants were predominantly Caucasian males with clinically diagnosed SAD[12]. Further work is needed to determine whether melatonin duration as a circadian signal of seasonality varies across ethnic groups with SAD.

The most significant limitation of this study was the use of a retrospective self-report measure of sleep duration. Self-reported sleep duration is not as accurate as more objective measures, such as polysomnographic recordings. A previous study found that when self-ratings were compared with objective measures, participants tended to overestimate sleep onset latency and underestimate their frequency of awakenings[42]. However, other studies have found acceptable levels of agreement between objective and subjective methods used to measure sleep[43,44,45]. In one of these studies, there was a positive correlation between objective and subjective measures of sleep duration ($r$ = 0.55)[43]. In addition, questionnaires and sleep diary reports of sleep duration have been reported to have strong, positive correlations[46]. Another potential limitation of this study was use of the SPAQ, a retrospective, self-report method for assessing seasonality. When estimating diagnosis of SAD and S-SAD, the SPAQ generates a high rate of false positives compared to structured clinical interviews that assess major depression[47]. The SPAQ, however, can reliably discriminate between individuals with clinically meaningful seasonality (i.e., SAD or S-SAD) and individuals with minimal seasonality[25], which is what was done in this study by combining SAD and S-SAD into the winter diagnosis group.

In summary, African and African American students with winter SAD or S-SAD reported significantly shorter sleep duration in winter than in summer. Considering that winter SAD is considered a condition with a preponderance of atypical symptoms of depression (with increased rather than decreased sleepiness), this is an unexpected finding that has implications for future research involving students and seasonality, and for the development of effective sleep hygiene strategies for students. It is possible that students with SAD/S-SAD reach an impasse between decreased motivation, energy, and concentration associated with winter depression and increased academic demands in winter (e.g., mid-term and final examinations) that results in sacrificing sleep duration and increasing wake duration to help compensate for decreased academic efficiency. It is also possible that typical symptoms of depression in winter SAD are more abundant than we previously thought in African and African American students, a possibility that will have to be explored in its own right. A limited awareness of SAD may contribute to the underdiagnosis and undertreatment of SAD[23]. Thus, concerted efforts are needed to study, treat, and increase awareness about seasonal changes in mood, behavior, and sleep in college students, and especially minority students and immigrants from lower latitudes, ultimately to improve mental and physical health, well-being, and academic performance.

## ACKNOWLEDGMENTS

This study was supported by the D.C. Department of Mental Health (Teodor T. Postolache, Principal Investigator). The authors appreciate the help of Joseph Soriano for commenting on an advanced copy of the manuscript and helping with formatting the final version of the manuscript.

## References

1. Borbely, A.A. (1982) A two process model of sleep regulation. *Hum. Neurobiol*. **1**, 195–204.
2. Kasper, S., Wehr, T.A., Bartko, J.J., Gaist, P.A., and Rosenthal, N.E. (1989) Epidemiological findings of seasonal changes in mood and behavior. A telephone survey of Montgomery County, Maryland. *Arch. Gen. Psychiatry* **46,** 823–833.
3. Rosen, L.N. and Rosenthal, N.E. (1991) Seasonal variations in mood and behavior in the general population: a factor-analytic approach. *Psychiatry Res.* **38,** 271–283.
4. Hardin, T.A., Wehr, T.A., Brewerton, T., Kasper, S., Berrettini, W., Rabkin, J., and Rosenthal, N.E. (1991) Evaluation of seasonality in six clinical populations and two normal populations. *J. Psychiatr. Res*. **25,** 75–87.
5. Wehr, T.A., Giesen, H.A., Schulz, P.M., Anderson, J.L., Joseph-Vanderpool, J.R., Kelly, K., Kasper, S., and Rosenthal, N.E. (1991) Contrasts between symptoms of summer depression and winter depression. *J. Affect. Disord*. **23**, 173–183.
6. Reynolds, C.F., 3rd and Kupfer, D.J. (1987) Sleep research in affective illness: state of the art circa 1987. *Sleep* **10,** 199–215.
7. Wehr, T.A. (1988) Sleep and biological rhythms in affective illness. In *Biological Rhythms and Mental Disorders.* Kupfer, D.J., Monk, T.A., and Barchas, J.D., Eds. Guilford Press, New York. pp. 143–175.
8. Anderson, J.L., Rosen, L.N., Mendelson, W.B., Jacobsen, F.M., Skwerer, R.G., Joseph-Vanderpool, J.R., Duncan, C.C., Wehr, T.A., and Rosenthal, N.E. (1994) Sleep in fall/winter seasonal affective disorder: effects of light and changing seasons. *J. Psychosom. Res.* **38,** 323–337.
9. Rosenthal, N.E., Sack, D.A., Gillin, J.C., Lewy, A.J., Goodwin, F.K., Davenport, Y., Mueller, P.S., Newsome, D.A. and Wehr, T.A. (1984) Seasonal affective disorder. A description of the syndrome and preliminary findings with light therapy. *Arch. Gen. Psychiatry* **41,** 72–80.
10. Shapiro, C.M., Devins, G.M., Feldman, B., and Levitt, A.J. (1994) Is hypersomnolence a feature of seasonal affective disorder? *J. Psychosom. Res*. **38(Suppl 1),** 49–54.
11. Aeschbach, D., Sher, L., Postolache, T.T., Matthews, J.R., Jackson, M.A., and Wehr, T.A. (2003) A longer biological night in long sleepers than in short sleepers. *J. Clin. Endocrinol. Metab.* **88**, 26–30.
12. Wehr, T.A., Duncan, W.C., Jr., Sher, L., Aeschbach, D., Schwartz, P.J., Turner, E.H., Postolache, T.T., and Rosenthal, N.E. (2001) A circadian signal of change of season in patients with seasonal affective disorder. *Arch. Gen. Psychiatry* **58**, 1108–1114.
13. Lingjaerde, O., Bratlid, T., Hansen, T., and Gotestam, K.G. (1986) Seasonal affective disorder and midwinter insomnia in the far north: studies on two related chronobiological disorders in Norway. In *Clinical Neuropharmacology*. Raven Press, New York. pp. 187–189.
14. Okawa, M., Shirakawa, S., Uchiyama, M., Oguri, M., Kohsaka, M., Mishima, K., Sakamoto, K., Inoue, H., Kamei, K., and Takahashi, K. (1996) Seasonal variation of mood and behaviour in a healthy middle-aged population in Japan. *Acta Psychiatr. Scand*. **94,** 211–216.
15. Buguet, A., Hankourao, O., and Gati, R. (1990) Self-estimates of sleep in African students in a dry tropical climate. *J. Environ. Psychol*. **10,** 363–369.
16. Bogui, P., Keita, M., Dah, C., Fidier, N., Buguet-Brown, M.L., and Buguet, A. (2002) [The sleep of Africans and Europeans in the Ivory Coast: questionnaire study]. *Sante* **12**, 263–270.
17. Montmayeur, A. and Buguet, A. (1992) Sleep patterns of European expatriates in a dry tropical climate. *J. Sleep Res*. **1,** 191–196.
18. Rohan, K.J. and Sigmon, S.T. (2000) Seasonal mood patterns in a northeastern college sample. *J. Affect. Disord*. **59**, 85–96.
19. Reid, A., Maldonado, C.C., and Baker, F.C. (2002) Sleep behavior of South African adolescents. *Sleep* **25,** 423–427.
20. Arakawa, M., Taira, K., Tanaka, H., Yamakawa, K., Toguchi, H., Kadekaru, H., Yamamoto, Y., Uezu, E., and Shirakawa, S. (2001) A survey of junior high school students' sleep habit and lifestyle in Okinawa. *Psychiatry Clin. Neurosci*. **55,** 211–212.
21. Brown, F.C., Buboltz, W.C., Jr., and Soper, B. (2002) Relationship of sleep hygiene awareness, sleep hygiene practices, and sleep quality in university students. *Behav. Med*. **28,** 33–38.
22. Hicks, R.A., Fernandez, C., and Pellegrini, R.J. (2001) The changing sleep habits of university students: an update. *Percept. Mot. Skills* **93**, 648.
23. Agumadu, C.O., Yousufi, S.M., Malik, I.S., Nguyen, M.C., Jackson, M.A., Soleymani, K., Thrower, C.M., Peterman,

M.J., Walters, G.W., Niemtzoff, M.J., Bartko, J.J., and Postolache, T.T. (2004) Seasonal variation in mood in African American college students in the Washington, D.C., metropolitan area. *Am. J. Psychiatry* **161**, 1084–1089.
24. Rosenthal, N.E., Bradt, G.H., and Wehr, T.A. (1984) Seasonal Pattern Assessment Questionnaire (SPAQ). National Institute of Mental Health, Bethesda, MD.
25. Magnusson, A. (1996) Validation of the Seasonal Pattern Assessment Questionnaire (SPAQ). *J. Affect. Disord*. **40**, 121–129.
26. Guzman, A., Rohan, K.J., Yousufi, S.M., Jackson, M.A., Nguyen, M.T., Soriano, J.J., and Postolache, T.T. (2007). Mood sensitivity to seasonal changes in African college students living in the greater Washington D.C. metropolitan area. *TheScientificWorldJOURNAL* **7**, 584-591.
27. Roberts, R.E., Roberts, C.R., and Chan, W. (2006) Ethnic differences in symptoms of insomnia among adolescents. *Sleep* **29**, 359–365.
28. Roberts, R.E., Roberts, C.R., and Chen, I.G. (2000) Ethnocultural differences in sleep complaints among adolescents. *J. Nerv. Ment. Dis*. **188**, 222–229.
29. Paine, S.J., Gander, P.H., and Travier, N. (2006) The epidemiology of morningness/eveningness: influence of age, gender, ethnicity, and socioeconomic factors in adults (30–49 years). *J. Biol. Rhythms* **21**, 68–76.
30. Durrence, H.H. and Lichstein, K.L. (2006) The sleep of African Americans: a comparative review. *Behav. Sleep Med*. **4,** 29–44.
31. Paine, S.J., Gander, P.H., Harris, R., and Reid, P. (2004) Who reports insomnia? Relationships with age, sex, ethnicity, and socioeconomic deprivation. *Sleep* **27**, 1163–1169.
32. Oginska, H. and Pokorski, J. (2006) Fatigue and mood correlates of sleep length in three age-social groups: school children, students, and employees. *Chronobiol. Int*. **23**, 1317–1328.
33. Steptoe, A., Peacey, V., and Wardle, J. (2006) Sleep duration and health in young adults. *Arch. Intern. Med*. **166**, 1689–1692.
34. Taheri, S., Lin, L., Austin, D., Young, T., and Mignot, E. (2004). Short sleep duration is associated with reduced leptin, elevated ghrelin, and increased body mass index. *PLoS Med.* **1,** e62.
35. Guzman, A., Zebrak, R., Rohan, K.J., Sumar, I.A., Stiller, J.W., Valadez-Meltzer, A., Olsen, C., Lapidus, M., Soriano, J.J., and Postolache, T.T. (2007) A prospective longitudinal study of seasonality in African students living in the greater Washington, D.C. metropolitan area. *TheScientificWorldJOURNAL* **7**, 557–583.
36. Tonetti, L., Barbato, G., Fabbri, M., Adan, A., and Natale, V. (2007) Mood seasonality: a cross-sectional study of subjects aged between 10 and 25 years. *J. Affect. Disord*. **97**, 155–160.
37. Natale, V., Adan, A., and Scapellato, P. (2005) Are seasonality of mood and eveningness closely associated? *Psychiatry Res*. **136**, 51–60.
38. Santos, C.B., Pratt, E.L., Hanks, C., McCann, J., and Craig, T.J. (2006) Allergic rhinitis and its effect on sleep, fatigue, and daytime somnolence. *Ann. Allergy Asthma Immunol*. **97,** 579–586; quiz 586–589, 671.
39. Fisher, L., Ghaffari, G., Davies, M., and Craig, T. (2005) Effects of poor sleep in allergic rhinitis. *Curr. Opin. Allergy Clin. Immunol*. **5**, 11–16.
40. Guzman, A., Tonelli, L.H., Roberts, D., Stiller, J.W., Jackson, M.A., Soriano, J.J., Yousufi, S., Rohan, K.J., Komarow, H., and Postolache, T.T. (2007) Mood-worsening with high-pollen-counts and seasonality: a preliminary report. *J. Affect. Disord*., in press.
41. Aeschbach, D., Postolache, T.T., Sher, L., Matthews, J.R., Jackson, M.A., and Wehr, T.A. (2001) Evidence from the waking electroencephalogram that short sleepers live under higher homeostatic sleep pressure than long sleepers. *Neuroscience* **102**, 493–502.
42. Baker, F.C., Maloney, S., and Driver, H.S. (1999) A comparison of subjective estimates of sleep with objective polysomnographic data in healthy men and women. *J. Psychosom. Res.* **47,** 335–341.
43. Akerstedt, T., Hume, K., Minors, D., and Waterhouse, J. (1994) The meaning of good sleep: a longitudinal study of polysomnography and subjective sleep quality. *J. Sleep Res*. **3,** 152–158.
44. Hauri, P.J. and Wisbey, J. (1992) Wrist actigraphy in insomnia. *Sleep* **15,** 293–301.
45. Lockley, S.W., Skene, D.J., and Arendt, J. (1999) Comparison between subjective and actigraphic measurement of sleep and sleep rhythms. *J. Sleep Res*. **8,** 175–183.
46. Patel, S.R., Malhotra, M.R., White, D.P., Hu, F.B., and Ayas, N.T. (2003) Validity and Reproducibility of Self-Reported Sleep Duration. Poster presentation at Associated Professional Sleep Societies 17th Annual Meeting, June 3–8, 2003, Chicago.
47. Raheja, S.K., King, E.A., and Thompson, C. (1996) The Seasonal Pattern Assessment Questionnaire for identifying seasonal affective disorders. *J. Affect. Disord*. **41**, 193–199.
